# Early‐life conditions impact juvenile telomere length, but do not predict later life‐history strategies or fitness in a wild vertebrate

**DOI:** 10.1002/ece3.8971

**Published:** 2022-06-20

**Authors:** Janske van de Crommenacker, Martijn Hammers, Hannah L. Dugdale, Terry A. Burke, Jan Komdeur, David S. Richardson

**Affiliations:** ^1^ 3647 Groningen Institute for Evolutionary Life Sciences (GELIFES) University of Groningen Groningen The Netherlands; ^2^ Faculty of Biological Sciences School of Biology University of Leeds Leeds UK; ^3^ Department of Animal and Plant Sciences University of Sheffield Sheffield UK; ^4^ 6106 School of Biological Sciences University of East Anglia Norfolk UK; ^5^ Nature Seychelles Roche Caiman Mahé Seychelles

**Keywords:** constraints, early‐life environmental conditions, future life span expectation hypothesis, juvenile telomere length, natural wild population, predictive adaptive responses, Seychelles warbler

## Abstract

Environmental conditions experienced during early life may have long‐lasting effects on later‐life phenotypes and fitness. Individuals experiencing poor early‐life conditions may suffer subsequent fitness constraints. Alternatively, individuals may use a strategic “Predictive Adaptive Response” (PAR), whereby they respond—in terms of physiology or life‐history strategy—to the conditions experienced in early life to maximize later‐life fitness. Particularly, the Future Lifespan Expectation (FLE) PAR hypothesis predicts that when poor early‐life conditions negatively impact an individual's physiological state, it will accelerate its reproductive schedule to maximize fitness during its shorter predicted life span. We aimed to measure the impact of early‐life conditions and resulting fitness across individual lifetimes to test predictions of the FLE hypothesis in a wild, long‐lived model species. Using a long‐term individual‐based dataset, we investigated how early‐life conditions are linked with subsequent fitness in an isolated population of the Seychelles warbler *Acrocephalus sechellensis*. How individuals experience early‐life environmental conditions may vary greatly, so we also tested whether telomere length—shorter telomers are a biomarker of an individual's exposure to stress—can provide an effective measure of the individual‐specific impact of early‐life conditions. Specifically, under the FLE hypothesis, we would expect shorter telomeres to be associated with accelerated reproduction. Contrary to expectations, shorter juvenile telomere length was not associated with poor early‐life conditions, but instead with better conditions, probably as a result of faster juvenile growth. Furthermore, neither juvenile telomere length, nor other measures of early‐life conditions, were associated with age of first reproduction or the number of offspring produced during early life in either sex. We found no support for the FLE hypothesis. However, for males, poor early‐life body condition was associated with lower first‐year survival and reduced longevity, indicating that poor early‐life conditions pose subsequent fitness constraints. Our results also showed that using juvenile telomere length as a measure of early‐life conditions requires caution, as it is likely to not only reflect environmental stress but also other processes such as growth.

## Graphical abstract

The Future Lifespan Expectation (FLE) hypothesis predicts that when poor early‐life conditions negatively impact an individual's physiological state, it will accelerate their reproductive schedule to maximize fitness during its shorter predicted life span. We measured the impact of early‐life conditions and resulting fitness across individual lifetimes to test the predictions of the FLE hypothesis in a wild, long‐lived model species, and we tested whether telomere length (TL) can provide an effective measure of the individual‐specific impact of early‐life conditions. We found no evidence for the FLE hypothesis, as shorter juvenile TL was not associated with poor early‐life conditions, but instead with better conditions. Neither juvenile TL, nor other measures of early‐life conditions, were associated with age of first reproduction or the rate of early‐life reproduction in either sex. Using juvenile TL as a measure of early‐life conditions requires caution, as it is likely to not only reflect environmental stress but also other processes such as growth.

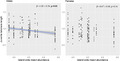

## 1 INTRODUCTION

Conditions experienced during early life may influence an individual's early‐life physiological state, but also have long‐lasting effects on phenotypes and fitness (Bateson et al., 2014; Monaghan, 2008). Poor early‐life conditions may negatively affect an individual's later‐life reproductive success and survival (Lindström, 1999; Lummaa & Clutton‐Brock, 2002; Nussey et al., 2007), while good early‐life conditions can have positive “Silver‐Spoon” fitness effects (Descamps et al., 2008; Marshall et al., 2017; Song et al., 2019) and delay late‐life reproductive senescence (Cooper & Kruuk, 2018). Such effects will greatly influence ecological and evolutionary dynamics within populations and are important to our understanding of life‐history evolution and population responses to climate change (Noble et al., 2018; Nussey et al., 2007; Uller, 2008).

One idea is that organisms may respond strategically to early‐life conditions through a “Predictive Adaptive Response” (PAR), to maximize later‐life fitness (Gluckman & Hanson, 2004; Gluckman et al., 2005). One potential PAR mechanism is outlined in the “Future Lifespan Expectation” (FLE) or “Internal PAR” hypothesis (Nettle et al., 2013). The FLE hypothesis predicts that a poor early‐life environment will have a long‐lasting negative impact on an individual's biological state (e.g., because of a reduced ability to invest in somatic tissue) resulting in reduced later‐life survival, irrespective of future environmental conditions (Nettle & Bateson, 2015; Nettle et al., 2013). In such a situation, individuals could benefit by adopting a life‐history strategy that maximizes fitness during their shorter predicted life span, that is, by accelerating their reproductive schedule (Nettle et al., 2013). This will be adaptive if it maximizes the fitness of individuals with poor early‐life state that face a reduced expected life span. This FLE hypothesis differs from the “Constraints” hypothesis (Gluckman & Hanson, 2004; Gluckman et al., 2005), which predicts no strategic response to poor early‐life conditions. Under the Constraints hypothesis, any later‐life phenotypic difference is the result of individuals experiencing poor early‐life conditions not being able to develop the optimal phenotype, and as such they are predicted to have reduced survival and reproductive success.

Direct tests of the FLE hypothesis are rare. A few studies appear consistent with this hypothesis, showing that poor early‐life external conditions are associated with an earlier age of first reproduction (Douhard et al., 2016; Nettle et al., 2010; Sloboda et al., 2009) or a higher investment in reproduction early in life (Cartwright et al., 2014), but others have found no signs of accelerated reproduction (Balbontín & Møller, 2015; Hayward et al., 2013). Furthermore, although fitness investments might differ considerably between males and females (Roff, 1992), most of these studies focused only on females and did not investigate sex‐specific effects in relation to the FLE hypothesis (Nager et al., 1999). Hence, research is needed that comprehensively measures variation in the impact of early‐life conditions and resulting fitness across individual lifetimes to test the predictions of the FLE hypothesis within natural populations of long‐lived species.

Quantifying the later‐life impact of early‐life conditions is not straightforward, especially in the wild. Measuring environmental conditions directly, for example, overall food availability, may seem like the obvious method to achieve this, but such measures may also be influenced by, and interact with, other environmental factors (e.g., ambient temperature), or by unknown variation within the variable itself (e.g., food quality). Moreover, the quantity and quality of food that different offspring receive may be determined by many individual‐specific variables (e.g., sibling competition, parental quality) that will not be accounted for by measuring overall food availability. Alternatively, one can measure a trait that is affected by the conditions that the individual has been exposed to. Body mass, size or condition may seem logical traits, but are likely to reflect energy reserves rather than intrinsic condition and are unlikely to be related to fitness in a linear fashion, especially in taxa like birds (Barnett et al., 2015; Bergeron et al., 2011). Instead, it would be better to measure a trait that reflects the intrinsic biological state of the individual (as a measure of the environmental impact it endured) (Kotrschal et al., 2007; Monaghan & Haussmann, 2006).

Telomeres—the noncoding nucleotide repeat structures that protect chromosome ends (Blackburn, 2000)—may be a suitable biomarker of an individual's intrinsic condition (Barrett et al., 2013; Haussmann et al., 2003; Monaghan & Haussmann, 2006). Telomere shortening is thought to be accelerated by oxidative stress (Epel, 2009; von Zglinicki, 2002) and induced by environmental stressors (Beckman & Ames, 1998). Consequently, telomere length—hereafter TL—may reflect previous exposure to factors that have impacted an individual's somatic state (Monaghan & Haussmann, 2006). Inherited variation in TL at birth (Dugdale & Richardson, 2018) and enhanced growth rate (accelerating telomere shortening through higher rates of cell division) are also factors shaping TL variation (Monaghan & Ozanne, 2018). However, juvenile telomere length (hereafter: JTL) may provide a useful indicator of the impact of early‐life conditions on an individual's biological state (Bateson et al., 2015; Cram et al., 2017).

Positive associations between early‐life TL and survival have been shown in the wild (e.g., Cram et al., 2017; Geiger et al., 2012; Lieshout et al., 2019; Watson et al., 2015) and in captivity (Heidinger et al., 2012). However, no telomere study to date has tested the PAR hypotheses or provided evidence of shorter TL being associated with accelerated reproduction, although a couple of studies did find longer early‐life TL to be associated with greater LRS in individuals that reached adulthood (Eastwood et al., 2018; Heidinger et al., 2021).

Here we test the use of juvenile telomere length (JTL) as a biomarker of the impact of early‐life environmental conditions and assess how this is linked to later‐life survival and reproduction in the Seychelles warbler *Acrocephalus sechellensis*. The Cousin Island population of this passerine is well suited to investigations of fitness‐related links under natural conditions: almost all birds have been followed throughout their lives (Hammers et al., 2015; Richardson et al., 2001) and the annual reproductive output and age of all individuals is known (Hammers et al., 2019; Raj Pant et al., 2020). Annual survival is not confounded with dispersal, as there is virtually no inter‐island migration (Komdeur et al., 2004). Hence estimates of fitness components are accurate and comprehensive. Telomeres appear to be suitable markers of biological state in this population because (i) considerable variation in TL exists (Sparks et al., 2021), and is positively related to food abundance (Spurgin et al., 2018), (ii) telomere shortening is linked to body condition (Barrett et al., 2013), and shorter (age‐controlled) TL and faster telomere attrition predict survival in adults (Barrett et al., 2013; Brown et al., 2021).

We assess key predictions of the FLE and Constraints hypotheses. Specifically, how early‐life state (measured as JTL) is linked to the following fitness‐related components: (i) age of first reproduction, (ii) initial reproductive output in early adult life, and (iii) life‐time reproductive success (LRS), in both males and females. We also assess the link between JTL and longevity, as both the Constraints and FLE hypotheses predict poor early‐life conditions to be associated with lower survival. However, under the FLE hypothesis, we also expect accelerated reproduction, while the Constraints hypothesis only predicts that shorter JTL will be associated with lower fitness overall. Testing these differing predictions in a wild population—where natural environmental conditions act upon individuals and fitness is realized under real‐life settings—will provide better understanding of the evolutionary and ecological importance of early‐life conditions in vertebrates.

## 2 MATERIALS AND METHODS

### 2.1 Study area, population, and data collection

The Seychelles warbler population on Cousin Island, Seychelles (29 ha; 04°20'S, 55°40'E) has been studied intensively since 1985 (Komdeur, 1994b; Richardson et al., 2003; van de Crommenacker et al., 2017). Since 1997, 96% of the population has been caught, individually marked with a combination of three color rings and a British Trust for Ornithology ring, and blood sampled (Richardson et al., 2001), either before fledgling (ca. day 14) or within the first year of life, and so are of known age. Seychelles warblers have a mean life expectancy from fledging of 5.5 years and maximum life span of 19 years (Hammers et al., 2015). Reproductive senescence starts at 6.5 years of age in females (Hammers et al., 2012) and 7.8 years in males (Raj Pant et al., 2020). All individuals are monitored throughout their lives allowing detailed measures of their environmental conditions (e.g., food availability, territorial group composition) and fitness. Inter‐island dispersal is virtually absent (<0.1%, Komdeur et al., 2004) and individual re‐sighting probability per season is very high (92 ± 0.02 <2 years—98 ± 0.01% for older birds, Brouwer et al., 2010). Therefore individuals not seen over two consecutive seasons can safely be assumed to be dead (Hammers et al., 2013).

Each year during the main breeding season (June–September), nearly all active nests are monitored to ascertain the breeding stage and social status of all birds within each of the ca. 110 territories. Monitoring has yielded a comprehensive set of behavioral, annual fitness, and life‐history parameters for nearly all birds. Sex and parentage have been determined using molecular techniques (Hadfield et al., 2006; Richardson et al., 2001; Sparks et al., 2021), resulting in a large pedigree database spanning 10 generations and containing nearly 1900 individuals (Sparks et al., 2021).

As Seychelles warblers are insectivorous (Komdeur, 1991), insect prey abundance determines the food availability. Island‐wide insect abundance—which shows considerable variation between years (van de Crommenacker et al., 2011)—was estimated each season as the mean number of insects per unit leaf area over all monthly surveys carried out on the island (Spurgin et al., 2018). A territory quality index was also calculated for all territories in all years following Komdeur (1992).

In our study population, cooperative breeding occurs as a result of a lack of suitable independent breeding vacancies (Komdeur, 1992). This drives some adults into becoming subordinates within a territory, where they either help with territory defense and the rearing of young (helpers), or not (non‐helpers; (Komdeur, 1994b)). Co‐breeding occurs: ca. 30%–40% of the subordinate females produce offspring in a given year (Hammers et al., 2019; Richardson et al., 2003). Extra‐group maternity does not occur, and extra‐pair paternity within the group is very rare (<1%), but extra‐group paternity is frequent (41%; Richardson et al., 2001; Raj Pant et al., 2019). Warblers on Cousin typically produce one clutch per season (Komdeur, 1998) normally of one egg (Richardson et al., 2001), thus assessments of early‐life environmental conditions are simplified, as single clutches are not confounded by sibling competition.

There is an established protocol for measuring TL in the Seychelles warbler (Barrett et al., 2012), with previous studies confirming that individual variation in TL acts as a biomarker of environmental stress in this species (Barrett et al., 2013; Bebbington et al., 2016, 2017; Spurgin et al., 2018). Thus, we have a dataset of individuals with their telomeres measured as a juvenile (JTL) for which we know all subsequent life history events and reproductive success until their death.

Field data from the main (July–September) and minor breeding periods (January–March) from 1996–2018 were used. JTL measures were available for individuals sampled in 1996–2014, and data on the offspring were produced by these individuals until September 2018. Morphological measurements including body mass and right tarsus length (±0.1 g and 0.1 mm, respectively) were available for each bird (Kingma et al., 2016). A blood sample (c. 25 μl) taken via brachial venipuncture was used to obtain DNA for molecular sexing, telomere, and parentage analyses. Parentage assignments with at least 0.8 probability were used to assign offspring to each focal bird (Sparks et al., 2021).

The used dataset included only birds for which TL was measured between 3–9 months of age—after the first 3‐month dependent period when telomere shortening was most rapid (Spurgin et al., 2018)—to capture the extent of telomere shortening (early environmental stress) just after development. Age at JTL measurement (in days) was determined based on calculated hatch date (Spurgin et al., 2018).

Fitness components included: (i) longevity, (ii) age of first reproduction, (iii) initial reproduction, and (iv) life‐time reproductive success (LRS). Longevity (years) was the last age at which an individual was observed, with at least two seasons of subsequent monitoring. We defined successful reproduction (hereafter “reproduction”) as producing an offspring that recruited into the adult population (survival of >1 year after hatching). In addition, we performed a sensitivity analysis by repeating the analyses but defining reproduction as producing an offspring that reached independence (age >3 months; Appendix Tables A5 and A6). Age of first reproduction was the age at which an individual was first assigned a genetic offspring. For the initial reproduction, we used the number of genetic offspring produced up to 3 years of age (the age by which most individuals that will gain a dominant position have done so; Hammers et al., 2013). LRS was defined as the total number of offspring an individual was assigned parentage during its life.

We performed all analyses for males and females separately as relationships between tarsus length, growth, and TL differ between the sexes in the Seychelles warbler (Brown et al., 2021; Spurgin et al., 2018). Moreover, sex‐specific differences in reproductive investments and parental care may affect the way in which early‐life conditions determine later‐life fitness. To avoid the data associated with JTL being influenced by individuals that die young and therefore have little opportunity to reproduce, only focal individuals that survived to at least 3 years of age were included in the “age of first reproduction” and “initial reproduction” data.

### 2.2 Telomere analyses

Relative (juvenile) TL was measured using qPCR as described in detail in Barrett et al. (2013) as part of another study by Spurgin et al. (2018). Briefly, each DNA sample was analyzed in duplicate/triplicate. LinRegPCR 2014.2 was used to correct baseline fluorescence, determine the window‐of‐linearity for each amplicon, and calculate individual well efficiencies. Threshold values (Nq) were set in the center of the window‐of‐linearity per amplicon for all samples. We corrected for variation across plates using a golden sample inter‐plate calibrator and calculated the TL for each sample as the amount of telomere DNA relative to that of a constantly expressed reference gene (GAPDH) that was simultaneously amplified on the same plate, following equation 1 in Pfaffl (2001). Based on the distribution of observed cq values, we excluded outlier samples with very large cq values (see Spurgin et al., 2018) which were assumed to be failed reactions. This work resulted in measurements for 1808 unique samples from juvenile and adult Seychelles warblers from 22 cohorts born between 1993 and 2014, including the measures used in the present study. Efficiencies (M ± SD) of the telomere and GAPDH reactions undertaken were 1.78 ± 0.05 and 1.92 ± 0.04, respectively. Intra‐plate repeatability was 0.74 (CI = 0.74, 0.75) and 0.73 (CI = 0.71, 0.74) for the GAPDH and Telomere Cq values, respectively. Inter‐plate repeatability of TL, based on 422 samples measured twice at different time points, was 0.68 (CI = 0.65, 0.70). For all other details, see Spurgin et al. (2018).

### 2.3 Statistical analyses

The overall dataset comprised 243 males and 273 females; the sample sizes for each specific test are given in the respective tables. Analyses were conducted using R v4.0.3 (R Core Team, 2018). Models were run either with a Poisson error distribution (with reproductive variables (offspring counts) as dependent) or a normal distribution (with JTL as dependent). JTL was the square root and z‐transformed (Verhulst, 2020) to improve linear model fits (Spurgin et al., 2018). As the age of JTL measurement was expected to be important for JTL, this variable was kept in the model (irrespective of its significance) when JTL was in the model. There were no repeated measurements, and where individuals were telomere‐sampled more than once as a juvenile only, the last measurement was used. All models were initially run as generalized linear mixed models (GLMM) in the lme4 v1.1‐21 package (Bates et al., 2014) with MotherID, CohortID (i.e., birth year) included as random variables. In the models with JTL as dependent variable (i.e., Table 1), qPCR plateID was also included as random variable to account for plate effects. For models with JTL as independent variable (Appendix Tables A2, A3, A4), we used the residuals of a linear regression between JTL ~plateID to account for plate effects. We checked for collinearity between fixed effects by calculating variance inflation factors (VIF) using the Performance v0.4.5 package in R; all VIFs were <3. Models were constructed separately for males and females, except for the very first model where we tested how JTL differed between the sexes controlling for all environmental conditions and individual‐specific variables mentioned in the following paragraph.

**TABLE 1:** Environmental and individual‐specific variables and their association with juvenile telomere length (JTL) in Seychelles warblers. Terms left in the final model are shown in bold. Terms significant in the analysis for the opposite sex are included in the final model, as is the term “age of JTL measurement” to control for age. All non‐retained variables were replaced back into the final model and their effect on that model and “t to remove” were reported. For the full model, see Appendix Table A1

Dependent = JTL	Males				Females			
	*n* = 181				*n* = 177			
	Estimate	SE	*t*	*p*	Estimate	SE	*t*	*p*
**Intercept**	**4.15**	**1.71**	**2.43**	.**02**	**0.56**	**0.88**	**0.64**	.**52**
**(log)island‐wide insect abundance**	**−1.02**	**0.38**	**−2.66**	.**01**	**−0.67**	**0.48**	**−1.42**	.**16**
**(log)age at JTL measurement**	**0.03**	**0.16**	**0.21**	.**83**	**−0.11**	**0.17**	**−0.7**	.**49**
Tarsus length	−0.01	0.08	−0.14	.89	−0.16	0.09	−1.75	.08
Body condition	0.06	0.08	0.74	.46	−0.01	0.07	−0.15	.88
Group size	−0.07	0.07	−1.04	.3	0.01	0.02	0.85	.4
(log)island‐wide insect abundance * tarsus length	−0.85	0.49	−1.76	.08	−0.03	0.11	−0.28	.78
Random effects								
MotherID	0.27 ± 0.52				0.27 ± 0.52			
CohortID	0.03 ± 0.17				0.03 ± 0.17			
PlateID	0.06 ± 0.24				0.06 ± 0.24			
Residual	0.44 ± 0.66				0.67 ± 0.82			
	R2 marginal: 0.04				R2 marginal: 0.01			
	R2 conditional: 0.47				R2 conditional: 0.44			

First, we tested to what extent JTL reflected the general measures of environmental conditions and individual‐specific variables during early life. We constructed a model with JTL as dependent variable, and (log) age (in days) at JTL measurement, island‐wide insect abundance, group size, tarsus length, and body mass as fixed variables. MotherID, CohortID, and PlateID were included here as random variables. The interaction between island‐wide insect abundance and tarsus length was tested as well to investigate the possibility that the relationship between island‐wide insect abundance and JTL depends on tarsus length.

When checking associations related to body mass, we kept tarsus length in the model so that body mass reflected body condition. We checked the importance of territory‐specific conditions in a separate model that included both island‐wide insect abundance and natal TQ (either or not corrected for group size, i.e., per capita TQ). Furthermore, we analyzed the relationships between food availability (island‐wide insect abundance) and tarsus length as well as body condition to check whether these followed the same pattern as the link between food availability and JTL.

Second, we tested whether JTL was linked with first‐year survival. These models had a binomial distribution with first‐year survival as the dependent variable, and (sqrt)JTL (included as the residual of (sqrt)JTL~plateID), (log) age at JTL measurement, per capita TQ (corrected for group size), tarsus length, and body mass as fixed independent variables. MotherID and CohortID were included as random variables.

Third, we tested whether JTL was associated with longevity (dependent) with insect prey abundance, (sqrt)JTL (included as the residual of (sqrt)JTL~plateID), (log)age of JTL measurement, and reproduction (yes/no) as fixed independent variables. MotherID and CohortID were included as random variables. We included whether they reproduced in their life, as costs of reproduction may reduce longevity (Lindén & Møller, 1989; Roff, 1992). However, it is important to note that a positive link between JTL and longevity cannot be regarded as conclusive evidence for the FLE hypothesis, as differences in longevity may be an effect of a PAR (i.e., accelerated reproduction) rather than a reason why it occurs. Only the life span that is anticipated by the individual at a young age should influence the FLE hypothesis.

Fourth, we tested the key prediction of the FLE hypothesis (i.e., acceleration of reproductive schedule in birds exposed to poor early‐life conditions) using models with either age of first reproduction or initial reproduction as dependent variable, and (sqrt)JTL (included as the residual of (sqrt)JTL~plateID) and (log) age of JTL measurement as fixed, independent variables. MotherID and CohortID were included as random variables. We also examined whether JTL was associated with LRS using the same predictor variables as above. We ran this model both with and without longevity included as a variable, because in long‐lived species, longevity is the most important predictor of LRS (Clutton‐Brock, 1988; Newton, 1989). Hence, we wanted to check whether any observed relationship between JTL and LRS was truly driven by their annual productivity or was mainly the result of individuals surviving longer. Individuals (*n* = 82) that were alive at the end of the study period were omitted from this analysis because LRS could not be calculated for these individuals.

We did not include environmental and social variables experienced by individuals during early life in the models testing associations between JTL and reproductive variables, as the idea is that early‐life JTL will be the accumulated result of these factors acting upon the focal individual, and thus reflect accumulated early‐life stress (Eastwood et al., 2018; Spurgin et al., 2018). The term “Age at JTL measurement” was also included in the analyses of survival/longevity, as age at JTL measurement (similar to age at the first catch) has been shown to be positively associated with first‐year survival (because fledglings have already survived the nestling stage (see Hammers et al., 2021)).

Lastly, we tested whether other early‐life variables (island‐wide insect abundance, tarsus length, body condition) were associated with either longevity or reproductive schedule. Models were made with either longevity, age of first reproduction, initial reproduction or LRS as the dependent variable, and island‐wide insect prey abundance, natal TQ, tarsus length, and body condition (mass with tarsus length as covariate) as fixed, independent variables. MotherID and CohortID were included as random variables.

Minimal model selection was based on stepwise backward elimination of nonsignificant terms (*p* > .05; in order of least significance). The final models (Table 1, Appendix Tables A2, A3, A4) contained the constant and all significant explanatory terms. In addition to showing the final models, the contribution of rejected terms to the final model was checked and the results of the full models including all explanatory variables are presented in the Appendix (following Whittingham et al., 2006). Eliminated terms were reintroduced in the final model to verify their nonsignificance. To compare model outcomes between males and females, we wanted to make sure that the final model structures for males and females were the same. Therefore, if a variable occurred in the final model for females but not in that for males (and vice versa), we still kept in it for the sake of comparison between the sexes. Statistical significance level was set at α = 0.05. To indicate the amount of variance explained by the model terms, each model summary shows the marginal R2 (the variance explained only by fixed effects) and the conditional R2 (the proportion of total variance explained through both fixed and random effects). For the binomial Model A2a, Tjur's R2 is given (which calculates the Coefficient of Discrimination D for generalized linear (mixed) models for binary outcomes) (Tjur, 2009). As we had no a priori reason to assume nonlinear relationships and as we did not want to overparameterize the models, quadratic terms were not tested. All model equations are given below the tables, and results from all full models are provided in the Appendix.

## 3 RESULTS

### 3.1 Associations between JTL and individual/environmental variables

**FIGURE 1:**
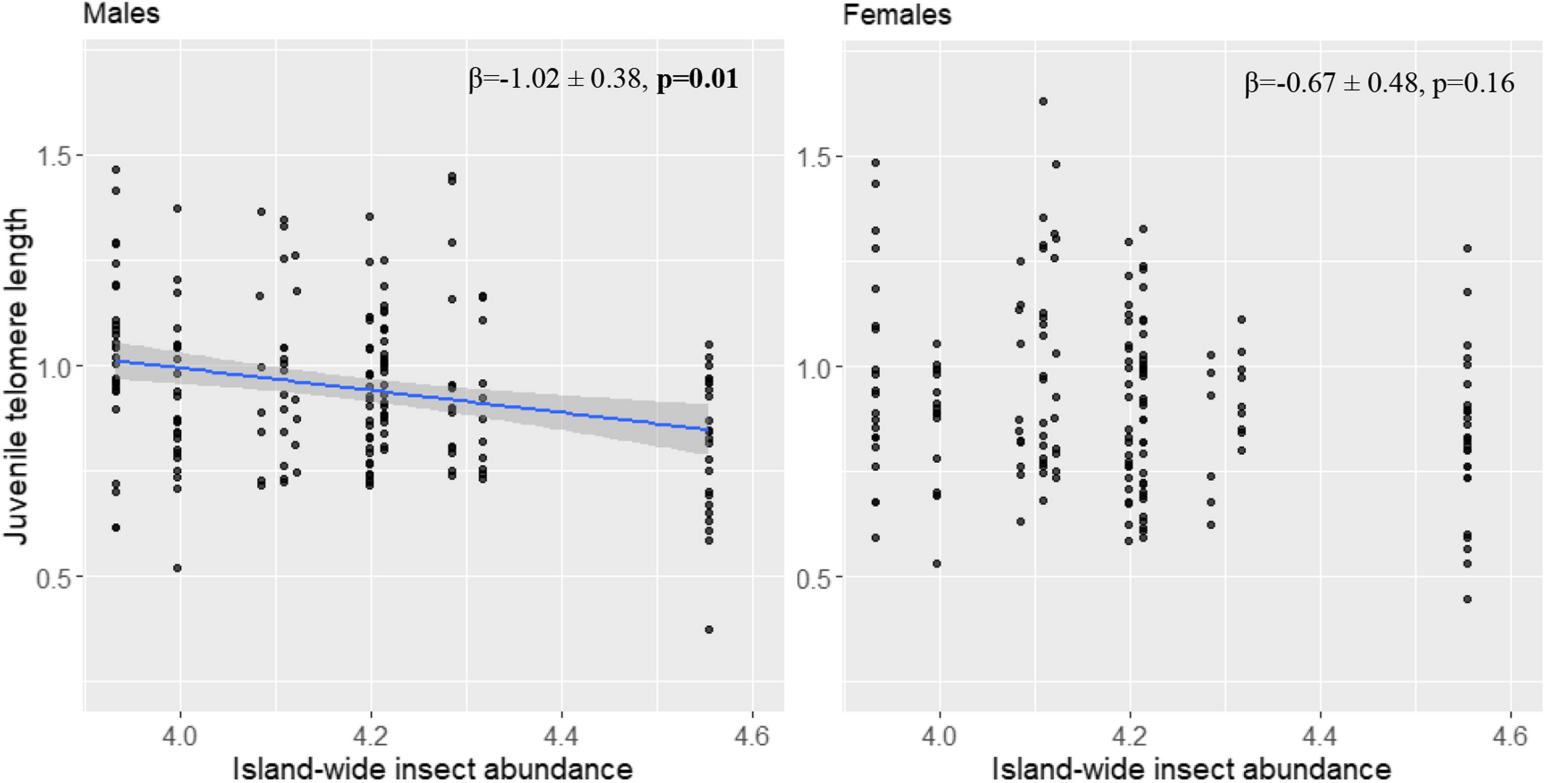
The relationship between juvenile telomere length (JTL) and island‐wide insect abundance in male (left panel) and female (right panel) Seychelles warblers. A linear regression line through the raw data is shown with 95% confidence intervals

Males had longer JTL than females (β = 0.08 ± 0.03, *p* = .009). There was no association between JTL and (log)age of JTL measurement for males (*p* = .83) or females (*p* = .49; Table 1). JTL was negatively associated with island‐wide insect abundance in males but not females (*p*_males_ = .010; *p*_females_ = .16, Table 1, Figure 1). JTL was not associated with tarsus length in either sex (*p*_males_ = .89 and *p*_females_ = .08, Table 1 (though this effect was borderline significant for females)), or with body condition or group size (all *p* > .30; Table 1), and there was no interaction between tarsus length and island‐wide insect abundance (*p* > .08; Table 1). The addition of natal TQ (either or not corrected for group size, i.e., per capita TQ) to the above‐mentioned model yielded similar results.

Tarsus length was positively associated with island‐wide insect abundance in females but not males (*p*_males_ = .58; *p*_females_ = .004), and body condition was positively associated with island‐wide insect abundance in both sexes (*p*_males_ = .005; *p*_females_ < .001).

### 3.2 Association between JTL and first‐year survival and longevity

**FIGURE 2:**
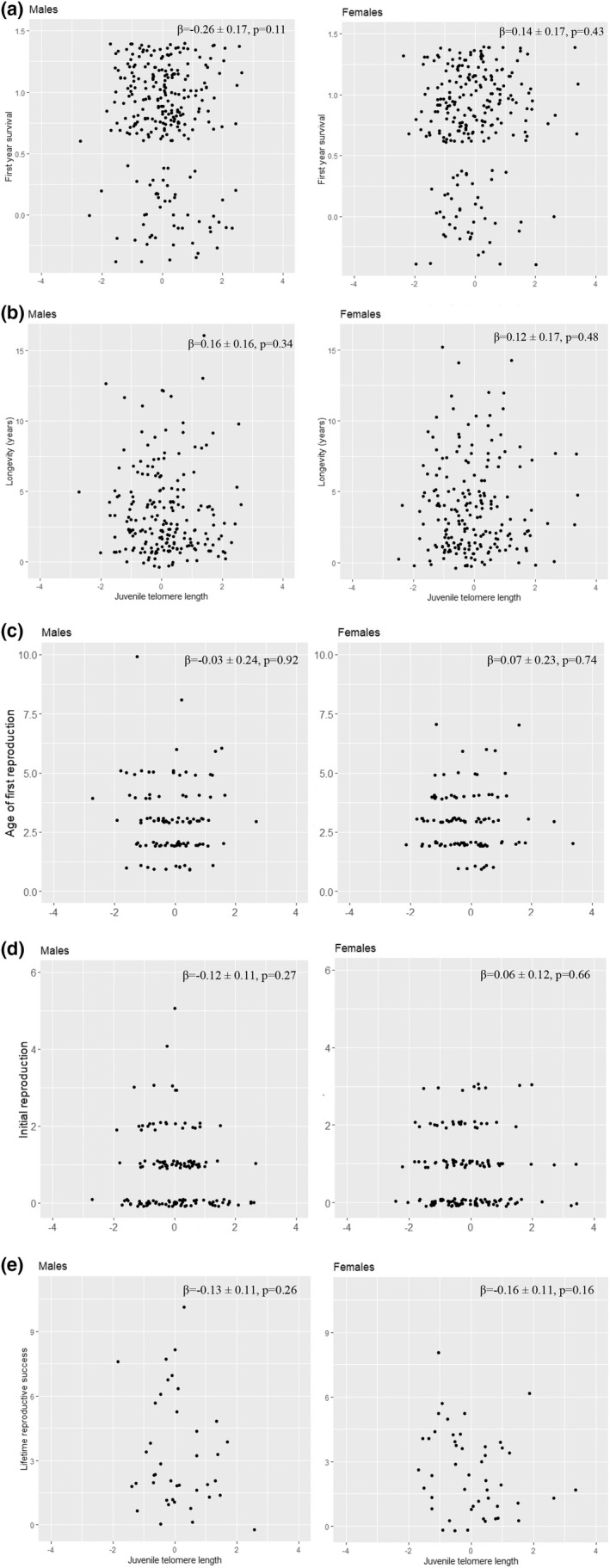
Associations between juvenile telomere length (JTL) and (a) survival to the first year, (b) longevity, (c) age of first reproduction, (d) initial reproduction, and (e) life‐time reproductive success (LRS) in male (left panels) and female (right panels) Seychelles warblers. The raw data are shown. For the model summaries (final and full models), see Appendix Tables A2, A3, A4

First‐year survival was 80% (males: 79.9% [207/259], females: 81.0% [192/237]). Neither JTL (*p*_males_ = .11; *p*_females_ = .43; Figure 2a), nor natal TQ (*p*_males_ = .52; *p*_females_ = .73) were associated with first‐year survival (Appendix Table A2a). Males had higher first‐year survival when body condition was higher (*p* = .012, Appendix Table A2a). For females, there was no such association (Appendix Table A2a).

Juvenile telomere length was not associated with longevity (Figure 2b), either without reproductive success (*p*_males_ = .30; *p*_females_ = .89) or with reproductive success added to the model (*p*_males_ = .34; *p*_females_ = .71, Appendix Table A2b).

### 3.3 JTL and reproductive schedule

In neither male nor female Seychelles warblers was JTL associated with age of first reproduction (*p*_males_ = .92; *p*_females_ = .74; Figure 2c, Appendix Table A3a), initial reproduction (*p*_males_ = .27; *p*_females_ = .66; Figure 2d, Appendix Table A3b) or LRS (*p*_males_ = .26 and *p*_females_ = .16; Figure 2e, Appendix Table A4). Repeating these analyses with reproduction defined as offspring that reached >3 months of age yielded similar results (Appendix Tables A5 and A6).

### 3.4 Other early‐life variables and longevity and reproductive schedule

We tested whether measures of early‐life conditions, other than JTL (i.e., island‐wide insect abundance, tarsus length, body condition) were linked with later life fitness. In males, a higher early‐life body condition was related with greater longevity (*p* < .001). In females, such a trend was also found, but this was not statistically significant (*p* = .08). A greater tarsus length was associated with shorter longevity in males but not in females (*p*_males_ = .001; *p*_females_ = .37). Island‐wide insect abundance was not associated with longevity (*p*_males_ = .16; *p*_females_ = .63). None of the early‐life variables (body condition, tarsus length or island‐wide insect abundance) were linked with either age of first reproduction (*p*_males_ > .24; *p*_females_ > .51), initial reproduction (*p*_males_ > .21; *p*_females_ > .25) or LRS (*p*_males_ > .10; *p*_females_ > .11). Repeating these analyses with per capita TQ instead of island‐wide insect abundance yielded similar results.

## 4 DISCUSSION

### 4.1 Associations between JTL and individual/environmental variables

To study the impact of early‐life environmental conditions on fitness in the Seychelles warbler, we first investigated the extent to which JTL reflected the measures of environmental conditions experienced during early life. Against our expectations, JTL was negatively (not positively) associated with island‐wide insect abundance (in males). This relationship implies that better conditions (more food availability) result in shorter JTL, but only in males. Good food resources during early life could potentially lead to more and/or faster growth (higher rates of cellular division) and consequently faster rates of telomere shortening, however, this is not necessarily the case as this link is far from straightforward (reviewed in Monaghan & Ozanne, 2018; also see Salmón et al., 2021; Vedder et al., 2017). The complexity of the link between growth/size and telomere shortening is further illustrated by the fact that the relationship between food availability (i.e., island‐wide insect abundance) and either tarsus length (positive and significant only for females) or body mass (positive for both sexes) differs from the negative relationship between island‐wide insect abundance and JTL (negative and significant only for males) as described above. From other studies, there is evidence that early‐life telomere dynamics/trajectories are more informative in relation to early‐life conditions than telomere length on its own (Boonekamp et al., 2014; Wood & Young, 2019). This might also be an alternative explanation for the lack of links between telomeres and fitness proxies in the present study. Further investigations using telomere dynamics rather than absolute length will be valuable to verify whether this is the case.

In Seychelles warblers, telomere shortening in males does not appear to harm their first‐year survival prospects (i.e., within the development phase itself), as we found no evidence of shorter JTL being linked with lower first‐year survival. This contrasts with studies on the king penguin *Aptenodytes patagonicus* and European storm petrel *Hydrobates pelagicus* that found shorter JTL to be associated with higher juvenile mortality (Geiger et al., 2012; Watson et al., 2015). We did find a higher body condition to be associated with an increased probability of reaching 1 year of age in males (supporting previous findings; Brown et al., 2022; Komdeur, 1994a).

Our result contrasts with a previous investigation which showed a positive relationship between food availability and TL in the Seychelles warbler (Spurgin et al., 2018). However, that study examined predominantly adults (of all ages) that had completed growth and maturation, where higher food availability may enable better self‐maintenance and thus less telomere loss. Interestingly, in zebra finches *Taeniopygia guttata*, a high quality diet did not affect JTL, but in adults, it reduced telomere shortening in females, but not in males (Noguera et al., 2015). These results emphasize that how environmental conditions are associated with telomere dynamics that differs between the sexes, as well as between age classes. This, and our own results, also underline that using JTL as a measure of early‐life conditions requires caution, as JTL not only reflects environmental stress but is also shaped by growth and other genetic or physiological processes, which are interrelated in a complex manner (Fagundes et al., 2013; Monaghan, 2014; Monaghan et al., 2009). Effects of growth and early‐life conditions can even interact to shape later‐life patterns of senescence (Tarry‐Adkins et al., 2009).

### 4.2 Association between JTL and longevity

Shorter JTL was not linked with longevity in either males or females. Longevity was greater for individuals that successfully reproduced at least once during their lifetime. This might seem surprising, as reproduction is expected to be costly (Lindén & Møller, 1989; Roff, 1992), but individuals that reproduce are probably of higher quality and thus may also survive longer. Furthermore, a likely explanation for this finding is that the birds that died as a juvenile were included in this analysis, and the individuals that reproduced also had a higher longevity because they survived the juvenile phase when mortality is relatively high (Brouwer et al., 2006). We found that greater tarsus length was associated with shorter longevity (only in males), suggesting that greater growth in early life may impede longevity later on. Why we found tarsus length but not JTL being linked with longevity needs further investigation. The variable “Age at JTL measurement” was correlated to subsequent longevity overall (when included along with reproductive success), which may be explained by juvenile birds sampled at older age having already survived for longer through the juvenile period (when mortality is relatively high; Brouwer et al., 2006), being probably more likely to survive further (Hammers et al., 2021).

### 4.3 JTL and reproductive schedule

Under the Future Lifetime Expectation (FLE) hypothesis, we predicted that shorter JTL (as a result of poor early‐life conditions) would be associated with an accelerated reproductive schedule. Our results did not follow these expectations and thus do not support the FLE hypothesis Firstly, shorter JTL was not associated with poor early‐life conditions, but the opposite. Secondly, there was no association between JTL and age of first reproduction, nor with higher initial reproduction, in either sex. In males though, shorter JTL was associated with better early‐life conditions (high food abundance). It is interesting to note that recent results from Seychelles warblers show that adult females, but not males, experience significant telomere shortening under greater stress (i.e., high reproductive effort, low food availability, hemoparasitic infection) (Brown et al., 2021). Perhaps, males can afford to start off in life with shorter telomeres as they are not as susceptible to telomere shortening due to stress in later life as are females.

We found no links between JTL and LRS (either with or without longevity included in the model). This suggests that having shorter telomeres as a juvenile (as a result of faster growth) does not impede later‐life reproductive performance. Our finding contrasts with those in collared flycatchers *Ficedula albicollis*, where good early‐life conditions increased female early‐life performance, but at a cost of faster reproductive aging and increased late‐life mortality (Spagopoulou et al., 2019). They also contrast from findings in wild purple‐crowned fairy‐wrens *Malurus coronatus*, where shorter early‐life TL was associated with lower LRS (Eastwood et al., 2018). In that study, the authors suggested that reduction in somatic maintenance during development (e.g., due to poor environmental conditions) particularly affects late‐life performance. However, they did not measure the early‐life food conditions, and so whether shorter JTL was reflective of poorer early‐life conditions could not be determined.

## 5 CONCLUSIONS

We aimed to determine whether measuring JTL would provide more resolution with which to assess the individual impact of early‐life conditions, and subsequently to better understand how early‐life circumstances are related to later life‐history strategies and fitness components in a wild vertebrate system. In contrast to our expectations, better early‐life conditions were linked with shorter (instead of longer) JTL. Our results do not provide support for the FLE hypothesis, as there was no evidence that poor early‐life conditions resulted in accelerated reproduction, and no link between JTL and life‐time reproductive success. In contrast, our results do support the Constraints hypothesis, at least for males, where better early‐life body condition was associated with greater first‐year survival and longevity. No associations between any metrics of early‐life condition and reproductive schedule were detected.

## AUTHOR CONTRIBUTION

**Janske van de Crommenacker:** Conceptualization (equal); Data curation (equal); Formal analysis (lead); Visualization (lead); Writing—original draft (lead); Writing—review & editing (lead). **Martijn Hammers:** Conceptualization (equal); Data curation (equal); Formal analysis (equal); Funding acquisition (supporting); Methodology (equal); Writing—review & editing (supporting). **Hannah Dugdale:** Conceptualization (equal); Data curation (equal); Formal analysis (supporting); Funding acquisition (equal); Methodology (supporting); Writing—review & editing (supporting). **Terry Burke:** Conceptualization (equal); Funding acquisition (equal); Writing—review & editing (supporting). **Jan Komdeur:** Conceptualization (equal); Funding acquisition (equal); Supervision (equal); Writing—review & editing (supporting). **David Richardson:** Conceptualization (lead); Data curation (equal); Formal analysis (supporting); Funding acquisition (equal); Methodology (equal); Supervision (lead); Writing—original draft (equal); Writing—review & editing (equal).

### OPEN RESEARCH BADGES

This article has earned an Open Data Badge for making publicly available the digitally‐shareable data necessary to reproduce the reported results. The data are available at https://doi.org/10.34894/5Y87OS.

## Data Availability

Data are deposited in the Dataverse repository through the University of Groningen (permalink https://doi.org/10.34894/5Y87OS).

## Appendix

**TABLE A1:** Environmental and individual‐specific variables and their association with juvenile telomere length (JTL) in Seychelles warblers. This table shows the full model, from which the final model in Table 1 is derived

Full model:								
JTL ~Environmental/individual variables	Males				Females			
	*n* = 181				*n* = 177			
	Estimate	SE	*t*	*p*	Estimate	SE	*t*	*p*
Intercept	−90.84	53.7	−1.69	.09	−41.25	56.5	−0.73	.47
(log)island‐wide insect abundance	21.77	12.8	1.7	.09	10.95	13.5	0.81	.42
(log)age at JTL measurement	−0.11	0.18	−0.61	.54	−0.24	0.21	−1.15	.25
Tarsus length	3.66	2.07	1.77	.08	1.8	2.3	0.78	.44
Body condition	0.06	0.08	0.75	.45	0.03	0.08	0.38	.71
Group size	−0.05	0.07	−0.78	.44	0.04	0.1	0.46	.65
(log)island‐wide insect abundance * tarsus length	−0.88	0.49	−1.78	.08	−0.47	0.55	−0.86	.39
Random effects								
MotherID	0.24 ± 0.49				0.09 ± 0.30			
CohortID	0.02 ± 0.13				0.05 ± 0.22			
PlateID	0.08 ± 0.28				0.32 ± 0.57			
Residual	0.46 ± 0.68				0.59 ± 0.77			
	R2 marginal: 0.07				R2 marginal: 0.04			
	R2 conditional: 0.48				R2 conditional: 0.46			

**TABLE A2:** Associations between juvenile telomere length (JTL) and (a) survival to the first year and (b) longevity in Seychelles warblers. The final and the full models are shown. Terms left in the final model are shown in bold. Terms significant in the analysis for the opposite sex are included in the final model, as is the term “age of JTL measurement” to control for age. All non‐retained variables were replaced back into the final model and their effect on that model and “t or z to remove” were reported

Final models:								
	Males				Females			
Dependent =	*n* = 168				*n* = 156			
Survival to the first year	Estimate	SE	*z*	*p*	Estimate	SE	*z*	*p*
**Intercept**	**1.37**	**6.04**	**0.23**	.**82**	**−1.28**	**6.54**	**−0.2**	.**85**
**Body condition**	**0.48**	**0.19**	**2.5**	.**012**	**0.16**	**0.19**	**0.85**	.**4**
**(log)age at JTL measurement**	**0.49**	**0.45**	**1.11**	.**27**	**0.83**	**0.47**	**1.77**	.**08**
JTL^a^	−0.26	0.17	−1.58	.11	0.14	0.17	0.78	.43
(log)TQ per capita	0.31	0.48	0.64	.52	−0.16	0.47	−0.34	.73
Tarsus length	−0.38	0.23	−1.65	.1	−0.15	0.25	−0.62	.53
	Tjur's R2: 0.05				Tjur's R2: 0.02			

Dependent =	*n* = 201				*n* = 197			
Longevity	Estimate	SE	*t*	*p*	Estimate	SE	*t*	*p*
**Intercept**	**−3.79**	**2.13**	**−1.78**	.**08**	**−4.51**	**2.37**	**−1.9**	.**06**
**(log)age at JTL measurement**	**1.15**	**0.42**	**2.74**	.**007**	**1.32**	**0.47**	**2.82**	.**005**
**Reproductive success**	**3.73**	**0.32**	**11.6**	**<.001**	**3.89**	**0.37**	**10.6**	**<.001**
JTL^a^	0.16	0.16	0.95	.34	0.12	0.17	0.7	.48
Random effects								
MotherID	0.11 ± 0.33				<0.001 ± <0.001			
CohortID	0.73 ± 0.85				0.23 ± 0.48			
Residual	4.56 ± 2.13				6.43 ± 2.54			
	R2 marginal: 0.46				R2 marginal: 0.39			
	R2 conditional: N/A				R2 conditional: N/A			

Full models								
	Males				Females			
Dependent =	*n* = 168				*n* = 156			
Survival to the first year	Estimate	SE	*z*	*p*	Estimate	SE	*z*	*p*
Intercept	2.75	8.42	0.33	.74	6.3	8.09	0.78	.44
JTL^a^	−0.39	0.25	−1.55	.12	0.07	0.22	0.3	.76
(log)age at JTL measurement	−0.35	0.69	−0.5	.61	0.9	0.58	1.55	.12
Body condition	0.76	0.27	2.77	.005	0.08	0.23	0.37	.71
*Per capita* (log)TQ	0.46	0.49	0.93	.35	−0.17	0.47	−0.36	.72
Tarsus length	−0.45	0.31	−1.46	.14	−0.43	0.3	−1.4	.16
	Tjur's R2: 0.08				Tjur's R2: 0.04			

Dependent =	*n* = 201				*n* = 197			
Longevity	Estimate	SE	*t*	*p*	Estimate	SE	*t*	*p*
Intercept	−3.72	2.12	−1.75	.08	−4.59	2.39	−1.92	.06
JTL^a^	0.16	0.16	0.95	.34	0.06	0.17	0.37	.71
(log)age at JTL measurement	1.13	0.42	2.7	.008	1.34	0.47	2.83	.005
Reproductive success	3.79	0.33	11.5	<.001	3.89	0.37	10.6	<.001
Random effects								
MotherID	<0.001 ± <0.001				<0.001 ± <0.001			
CohortID	0.72 ± 0.85				<0.001 ± <0.001			
Residual	4.56 ± 2.13				6.46 ± 2.54			
	R2 marginal: 0.51				R2 marginal: 0.39			
	R2 conditional: 0.42				R2 conditional: N/A			

^a^JTL is fitted in the models as Residual((sqrt)JTL~PlateID).

**TABLE A3:** Associations between juvenile telomere length (JTL) and reproductive parameters that test the FLE hypothesis in Seychelles warblers: (a) age of first reproduction and (b) initial reproduction. The final and the full models are shown. Terms left in the final model are shown in bold. Terms significant in the analysis for the opposite sex are included in the final model, as is the term “age of JTL measurement” to control for age. All non‐retained variables were replaced back into the final model and their effect on that model and “t to remove” were reported

Final models:								
	Offspring >1 year							
	Males				Females			
Dependent =	*n* = 102				*n* = 103			
a) Age of first reproduction	Estimate	SE	*t*	*p*	Estimate	SE	*t*	*p*
**Intercept**	**1.69**	**2.02**	**0.84**	.**4**	**0.65**	**1.64**	**0.4**	.**69**
**(log)age at JTL measurement**	**0.27**	**0.39**	**0.71**	.**48**	**0.45**	**0.32**	**1.42**	.**16**
JTL^a^	−0.03	0.24	−1.11	.92	0.07	0.23	0.33	.74
Random effects								
MotherID	0.91 ± 0.95				0.12 ± 0.34			
CohortID	0.94 ± 0.97				<0.001 ± <0.001			
Residual	0.91 ± 0.95				1.47 ± 1.21			
	R2 marginal: 0.004				R2 marginal: 0.02			
	R2 conditional: 0.67				R2 conditional: N/A			

	Males				Females			
Dependent =	*n* = 142				*n* = 148			
b) Initial reproduction	Estimate	SE	*t*	*p*	Estimate	SE	*t*	*p*
**Intercept**	**1.26**	**0.99**	**1.26**	.**21**	**2.88**	**0.99**	**2.9**	.**004**
**(log)age at JTL measurement**	**−0.09**	**0.19**	**−0.48**	.**63**	**−0.41**	**0.19**	**−2.14**	.**034**
JTL^a^	−0.12	0.11	−1.11	.27	0.06	0.12	0.45	.66
Random effects								
MotherID	0.40 ± 0.63				0.11 ± 0.34			
CohortID	0.16 ± 0.40				<0.001 ± <0.001			
Residual	0.37 ± 0.61				0.70 ± 0.83			
	R2 marginal: 0.60				R2 marginal: 0.39			
	R2 conditional: 0.001				R2 conditional: N/A			

Full models:								
	Offspring >1 year							
	Males				Females			
Dependent =	*n* = 102				*n* = 103			
a) Age of first reproduction	Estimate	SE	*t*	*p*	Estimate	SE	*t*	*p*
Intercept	1.72	2.04	0.84	.4	0.63	1.64	0.39	.7
JTL^a^	−0.03	0.24	−1.11	.92	0.07	0.23	0.33	.74
(log)age at JTL measurement	0.27	0.39	0.69	.49	0.46	0.32	1.42	.16
Random effects								
MotherID	0.91 ± 0.95				0.13 ± 0.36			
CohortID	0.94 ± 0.97				<0.001 ± <0.001			
Residual	0.93 ± 0.96				1.47 ± 1.21			
	R2 marginal: 0.004				R2 marginal: 0.02			
	R2 conditional: 0.67				R2 conditional: N/A			

	Males				Females			
Dependent =	*n* = 142				*n* = 148			
b) Initial reproduction	Estimate	SE	*t*	*p*	Estimate	SE	*t*	*p*
Intercept	1.22	1	1.22	.23	2.86	0.99	2.88	.005
JTL^a^	−0.12	0.11	−1.11	.27	0.06	0.12	0.45	.66
(log)age at JTL measurement	−0.09	0.19	−0.44	.66	−0.41	0.19	−2.11	.036
Random effects								
MotherID	0.36 ± 0.60				0.11 ± 0.33			
CohortID	0.16 ± 0.40				<0.001 ± <0.001			
Residual	0.38 ± 0.40				0.70 ± 0.84			
	R2 marginal: 0.008				R2 marginal: 0.04			
	R2 conditional: 0.57				R2 conditional: N/A			

^a^ JTL is fitted in the models as Residual((sqrt)JTL~PlateID).

**TABLE A4:** The association between juvenile telomere length (JTL) and life‐time reproductive success (LRS) in Seychelles warblers. The final and the full models are shown. Terms left in the final model are shown in bold. Terms significant in the analysis for the opposite sex are included in the final model, as is the term “age of JTL measurement” to control for age. All non‐retained variables were replaced back into the final model and their effect on that model and “z to remove” were reported

Final model:								
	Offspring >1 year							
	Males				Females			
Dependent =	*n* = 40				*n* = 48			
Life‐time reproductive success	Estimate	SE	*z*	*p*	Estimate	SE	*z*	*p*
**Intercept**	**0.98**	**0.15**	**6.35**	**<.001**	**0.77**	**0.13**	**5.8**	**<.001**
**Longevity**	**0.5**	**0.19**	**2.63**	.**009**	**0.14**	**0.22**	**0.66**	.**51**
**(log)age at JTL measurement**	**0.2**	**0.22**	**0.93**	.**35**	**0.31**	**0.23**	**1.35**	.**18**
JTL^a^	−0.13	0.11	−1.12	.26	−0.16	0.11	−1.42	.16
Random effects								
MotherID	<0.001 ± <0.001				0.18 ± 0.43			
CohortID	0.13 ± 0.37				<0.001 ± <0.001			
	R2 marginal: 0.20				R2 marginal: 0.07			
	R2 conditional: N/A				R2 conditional: N/A			

Full model:								
	Offspring >1 year							
	Males				Females			
	*n* = 40				*n* = 48			
Life‐time reproductive success	Estimate	SE	*z*	*p*	Estimate	SE	*z*	*p*
Intercept	0.99	0.15	6.57	<.001	0.79	0.13	6.11	<.001
JTL^a^	−0.13	0.11	−1.12	.26	−0.16	0.11	−1.42	.16
(log)age at JTL measurement	0.12	0.23	0.54	.59	0.24	0.23	1.04	.3
Longevity	0.53	0.21	2.57	.01	0.14	0.21	0.65	.52
Random effects								
MotherID	0.006 ± 0.08				0.15 ± 0.39			
CohortID	0.11 ± 0.33				<0.001 ± <0.001			
	R2 marginal: 0.17				R2 marginal: 0.13			
	R2 conditional: 0.40				R2 conditional: N/A			

^a^ JTL is fitted in the models as Residual((sqrt)JTL~PlateID).

### 1

Sensitivity tests: alternative models with successful reproduction being defined as the production of offspring that reached fledgling stage (age >3 months). We performed this test with a different definition of reproduction, as there may be differences in the factors affecting offspring survival: the probability of offspring surviving the first 3 months is mostly dependent of parental provisioning, whereas the recruitment of offspring (surviving the first year) is mostly dependent on how the young perform themselves after reaching independence at ca. 3 months of age.

**TABLE A5:** The association between juvenile telomere length (JTL) and reproductive parameters that test predictions from the FLE hypothesis in Seychelles warblers: (a) age of first reproduction and (b) initial reproduction. In this sensitivity test, successful reproduction was defined as the production of offspring that reached fledgling stage (age >3 months). The final and the full model are shown. Terms left in the final model are shown in bold. Terms significant in the analysis for the opposite sex are included in the final model, as is the term “age of JTL measurement” to control for age. All non‐retained variables were replaced back into the final model and their effect on that model and “t to remove” were reported

Final models:								
	Offspring >3 months							
	Males				Females			
Dependent =	*n* = 105				*n* = 106			
(a) Age of first reproduction	Estimate	SE	*t*	*p*	Estimate	SE	*t*	*p*
**Intercept**	**2.29**	**1.82**	**1.26**	.**21**	**1.55**	**1.48**	**1.05**	.**3**
**(log)age at JTL measurement**	**0.12**	**0.35**	**0.35**	.**73**	**0.24**	**0.29**	**0.85**	.**4**
JTL^a^	0.02	0.22	0.11	.91	0.12	0.2	0.58	.56
Random effects								
MotherID	0.93 ± 0.96				<0.001 ± <0.001			
CohortID	0.65 ± 0.81				<0.001 ± <0.001			
Residual	0.64 ± 0.80				1.35 ± 1.16			

	Males				Females			
Dependent =	*n* = 142				*n* = 148			
(b) Initial reproduction	Estimate	SE	*t*	*p*	Estimate	SE	*t*	*p*
**Intercept**	**1.48**	**1.14**	**1.3**	.**2**	**3.23**	**1.2**	**2.7**	.**008**
**(log)age at JTL measurement**	**−0.1**	**0.22**	**−0.44**	.**66**	**−0.44**	**0.23**	**−1.89**	.**06**
JTL^a^	−0.15	0.13	−1.17	.25	0.13	0.15	0.89	.37
Random effects								
MotherID	0.58 ± 0.76				<0.001 ± <0.001			
CohortID	0.19 ± 0.43				0.03 ± 0.16			
Residual	0.46 ± 0.70				1.11 ± 1.05			

Full models:								
	Offspring >3 months							
	Males				Females			
Dependent =	*n* = 105				*n* = 106			
(a) Age of first reproduction	Estimate	SE	*t*	*p*	Estimate	SE	*t*	*p*
Intercept	2.27	1.83	1.24	.22	1.49	1.49	1	.32
(log)age at JTL measurement	0.12	0.35	0.35	.73	0.26	0.29	0.88	.38
JTL^a^	0.02	0.22	0.11	.91	0.12	0.2	0.58	.56
Random effects								
MotherID	0.93 ± 0.96				<0.001 ± <0.001			
CohortID	0.63 ± 0.80				<0.001 ± <0.001			
Residual	0.64 ± 0.80				1.35 ± 1.16			

	Males				Females			
Dependent =	*n* = 142				*n* = 148			
(b) Initial reproduction	Estimate	SE	*t*	*p*	Estimate	SE	*t*	*p*
Intercept	1.43	1.14	1.25	.21	3.19	1.2	2.67	.009
(log)age at JTL measurement	−0.09	0.22	−0.4	.69	−0.43	0.23	−1.85	.07
JTL^a^	−0.15	0.13	−1.17	.25	0.13	0.15	0.89	.37
Random effects								
MotherID	0.54 ± 0.74				<0.001 ± <0.001			
CohortID	0.18 ± 0.43				0.02 ± 0.15			
Residual	0.46 ± 0.68				1.11 ± 1.06			

^a^ JTL is fitted in the models as Residual((sqrt)JTL~PlateID).

**TABLE A6:** The association between juvenile telomere length (JTL) and life‐time reproductive success in Seychelles warblers. In this sensitivity test, successful reproduction was defined as the production of offspring that reached fledgling stage (age >3 months). The final and the full model are shown. Terms left in the final model are shown in bold. Terms significant in the analysis for the opposite sex are included in the final model, as is the term “age of JTL measurement” to control for age. All non‐retained variables were replaced back into the final model and their effect on that model and “z to remove” were reported

Final model:								
	Offspring >3 months							
	Males				Females			
Dependent =	*n* = 40				*n* = 48			
Life‐time reproductive success	Estimate	SE	*z*	*p*	Estimate	SE	*z*	*p*
**Intercept**	**1.12**	**0.15**	**7.5**	**<.001**	**1.01**	**0.13**	**7.91**	**<.001**
**(log)age at JTL measurement**	**0.45**	**0.21**	**2.17**	.**03**	**0.43**	**0.22**	**1.96**	.**05**
**Longevity**	**0.51**	**0.17**	**2.94**	.**003**	**0.31**	**0.2**	**1.54**	.**12**
JTL^a^	−0.08	0.1	−0.77	.44	−0.08	0.1	−0.78	.44
Random effects								
MotherID	<0.001 ± <0.001				0.16 ± 0.40			
CohortID	0.13 ± 0.36				0.02 ± 0.14			

Full model:								
	Offspring >3 months							
	Males				Females			
Dependent =	*n* = 40				*n* = 48			
Life‐time reproductive success	Estimate	SE	*z*	*p*	Estimate	SE	*z*	*p*
Intercept	1.12	0.15	7.67	<.001	1.02	0.13	8.14	<.001
(log)age at JTL measurement	0.4	0.22	1.81	.07	0.4	0.23	1.77	.08
Longevity	0.53	0.19	2.78	.005	0.3	0.2	1.54	.12
JTL^a^	−0.08	0.1	−0.77	.44	−0.08	0.1	−0.78	.44
Random effects								
MotherID	0.004 ± 0.06				0.15 ± 0.39			
CohortID	0.12 ± 0.34				0.01 ± 0.12			

^a^ JTL is fitted in the models as Residual((sqrt)JTL~PlateID).
